# An evaluation of health systems equity in Indonesia: study protocol

**DOI:** 10.1186/s12939-018-0822-0

**Published:** 2018-09-12

**Authors:** Virginia Wiseman, Hasbullah Thabrany, Augustine Asante, Manon Haemmerli, Soewarta Kosen, Lucy Gilson, Anne Mills, Andrew Hayen, Viroj Tangcharoensathien, Walaiporn Patcharanarumol

**Affiliations:** 1 Department of Global Health and Development London School of Hygiene & Tropical Medicine, London, UK; 2 Kirby Institute, University of New South Wales, Sydney, Australia; 3 Centre for Health Economics and Policy Studies, University of Indonesia, Jakarta, Indonesia; 4 School of Public Health & Community Medicine, University of New South Wales, Sydney, Australia; 5 National Institute of Health Research & Development, Jakarta, Indonesia; 6 School of Public Health and Family Medicine, University of Cape Town, Cape Town, South Africa; 7 Faculty of Health, University of Technology Sydney, Sydney, Australia; 8 International Health Policy Program, Ministry of Public Health, Nonthanburi, Thailand

**Keywords:** Universal health coverage, Financing, Equity, Benefit incidence, Financing incidence, Catastrophic health spending, Impoverishing health spending

## Abstract

**Background:**

Many low and middle income countries are implementing reforms to support Universal Health Coverage (UHC). Perhaps one of the most ambitious examples of this is Indonesia’s national health scheme known as the JKN which is designed to make health care available to its entire population of 255 million by end of 2019. If successful, the JKN will be the biggest single payer system in the world. While Indonesia has made steady progress, around a third of its population remains without cover and out of pocket payments for health are widespread even among JKN members. To help close these gaps, especially among the poor, the Indonesian government is currently implementing a set of UHC policy reforms that include the integration of remaining government insurance schemes into the JKN, expansion of provider networks, restructuring of provider payments systems, accreditation of all contracted health facilities and a range of demand side initiatives to increase insurance uptake, especially in the informal sector. This study evaluates the equity impact of this latest set of UHC reforms.

**Methods:**

Using a before and after design, we will evaluate the combined effects of the national UHC reforms at baseline (early 2018) and target of JKN full implementation (end 2019) on: progressivity of the health care financing system; pro-poorness of the health care delivery system; levels of catastrophic and impoverishing health expenditure; and self-reported health outcomes. In-depth interviews with stakeholders to document the context and the process of implementing these reforms, will also be undertaken.

**Discussion:**

As countries like Indonesia focus on increasing coverage, it is critically important to ensure that the poor and vulnerable - who are often the most difficult to reach – are not excluded. The results of this study will not only help track Indonesia’s progress to universalism but also reveal what the UHC-reforms mean to the poor.

## Introduction

Concerns about the poor and most vulnerable not getting adequate access to quality health care are widespread in low and middle-income countries (LMICs) and have led to an intense advocacy for universal health coverage (UHC). Equity, defined by the World Health Organization as ‘the absence of avoidable or remediable differences among groups of people, whether those groups are defined socially, economically, demographically, or geographically’ [1] - is fundamental to UHC. However, emerging evidence is showing that without adequate focus on the measurement of equity, vulnerable populations may continue to receive inadequate or inferior health care [2].

Financial barriers are a major hindrance to accessing quality health services [3–5]. The World Health Report 2000 emphasises that a key dimension of a health system’s performance is the fairness of its financing system [1]. Globally, some 100 million people fall below the poverty line every year as a result of out-of-pocket expenditures on health, and a further 1.2 billion, already living in poverty, are pushed deeper into it [1]. In countries such as Pakistan, Laos, The Philippines, Bangladesh, Indonesia and Vietnam, out-of-pocket payments represent around 50% or more of total health expenditure [1]. Moreover, some countries reported to have achieved universal coverage by prepayment schemes, such as China and Brazil, still experience high prevalence of catastrophic health spending and medical impoverishment [6, 7].

UHC has been defined by the 2005 World Health Assembly as “access to key promotive, preventive, curative and rehabilitative health interventions for all at an affordable cost, thereby achieving equity in access” [8]. Effective implementation of UHC requires equity in health care, defined as payment for health services according to capacity to pay and the receipt of benefits according to need [9]. This implies that the allocation of government health spending needs to be focused on the poor, and recognises differences in the cost of accessing health care by different geographic, demographic and socio-economic groups. There is evidence that primary health care is pro-poor, suggesting a greater investment in these services, along with the removal of barriers to accessing care, can enhance equity [10]. In many LMICs, however, government health spending tends to concentrate on inpatient hospital services, most of which is urban-based and often too costly to be accessed by the poor [10].

A pro-poor publicly financed health-care system is particularly important given the growing pluralism of health-care systems in LMICs [11]. Households in LMICs use a wide range of public and private health-care providers, many of whom are not regulated by national health authorities [12] and may be paid for directly by out-of-pocket payments [13]. Such direct payments affect the poor more than the rich and tax financed health-care may protect the most vulnerable against the risk of financial catastrophe in times of illness [14, 15]. Dual practice – whereby health workers combine salaried, public-sector clinical work with a fee-for-service private clientele - is common in LMICs such as Indonesia and is reported to play a key role in undermining access to public services, especially by the poor [16]. Other motivations for universal health-care include redressing historical inequities in the distribution of health-care, reducing health inequality and raising the human capital of the poor and thereby the growth potential of the economy [17]. Governments worldwide are seeking to develop their health financing systems in ways that ensure - and, critically, sustain - universal coverage [18, 19].

### The Indonesian context

Indonesia is a lower middle-income country with a Gross National Income (GNI) per capita of US$3630 [20] with high Gross Domestic Product (GDP) growth, averaging 5.6% between 2007 and 2016 [21]. It is the third most populous country in Asia and the fourth largest in the world with around 255 million people [20]. Like other LMICs, Indonesia faces significant challenges in the health sector despite notable progress in the past decades especially in relation to improved life expectancy. Indonesia’s maternal mortality ratio (MMR) remains one of the highest in Southeast Asia, estimated at 359 per 100,000 live births in 2012 [22]; this is significantly higher than the MMR in neighbouring countries - Malaysia (29 per 100,000 in 2013) and Thailand (26 per 100,000 in 2013) [23]. With neonatal mortality remaining high at 19 per 1000 live births [21], Indonesia has the 8th highest number of neonatal deaths in the world and large disparities between the wealthiest (10 neonatal deaths per 1000 live births) and poorest quintiles (29 per 1000) [23, 24]. Malnutrition is a major problem with around 37% (8.4 million) of children under five years being stunted while overweight and obesity in adults has doubled in the past decade [25, 26]. Indonesia also faces a double burden of disease characterised by rising non-communicable diseases and a high incidence of communicable diseases [27].

Underpinning these problems are significant disparities in access to quality health services across geographic regions and socioeconomic groups. For example, health outcomes are lower in many Eastern Indonesian provinces as well as in rural areas and among people from the lowest wealth quintile [22]. The child mortality rate is less than 10 per 1000 live births in most provinces of Java and Sumatera but the rate is 2.5 times higher in the Eastern province of Maluku and North Maluku [22]. Rural households are reported to have an under-five mortality rate one-third higher than that in urban households [22]. High government funding allocations to hospitals (less frequently utilised by poor and disadvantaged communities) and elevated government spending on pharmaceuticals has also reduced investment in primary and promotive health services [27]. Indonesia spends only slightly more than 2% of its GDP on health, approximately half the level of other comparable income countries [28]. About half of all health spending is public and one-third comes directly from of out of pocket payments by households [28].

A key response by the Government has been the development of a compulsory national health insurance scheme designed to pave the way for the achievement of universal coverage [29]. This scheme, known as *Jaminan Kesehatan Nasional* (JKN), seeks to make comprehensive care available to the entire population by 2019. The JKN brings together all major health insurance schemes (*Askes, Jamkesmas, Jamsostek and Jamkesda*) under a single agency - the Social Security Management Corporation for the Health Sector (BPJS Kesehatan) [30]. Prior to this, Indonesian healthcare was highly fragmented with private insurance schemes for those who could afford it, basic state provision for the very poorest, and NGOs in specialised areas providing support in between. Through the JKN, the Indonesian Government sought to improve the situation for the ‘missing middle’, those citizens too poor to afford health insurance but deemed not poor enough for government support (7).

Indonesia has made steady progress with around 165 million people now members of the JKN, making it the biggest single-payer health system in the world [31]. There is however mounting evidence of areas where the JKN is underperforming and without action, the JKN is unlikely to reach expected levels of population coverage, service coverage or financial protection by 2019. It is estimated that 90 million (40% of the population) remain uncovered, most of these working in the informal sector [32]. JKN members continue to incur high out-of-pocket health expenditures [33]. Moreover, Indonesia’s public health financing remains at roughly half the estimated requirement for UHC [32].

Responding to the current challenges facing the JKN, the Indonesian government is initiating and strengthening several important reforms ranging from re-structuring provider payment schemes through to socialization campaigns to raise awareness of the scheme and its benefits [34]. Strategies for increasing fiscal space for health through increasing tobacco tax and the phasing out of subsidies on fuel are also proposed [30]. Our study investigates the equity impact of this latest phase of UHC-reforms that are designed to provide affordable health care to all citizens by 2019.

## Research objectives

The over-arching goal of this study is to assess the equity impact of the most recent package of UHC reforms implemented by the Indonesian government to support universal coverage. Specific study objectives are to:

1. Measure and compare key equity outcomes - including health care utilisation, subsidies received through the use of health services, payments people make for health care, andself-assessed health – in early 2018 (study baseline) and end of 2019 (target of JKN full implementation);
2. Develop and apply ‘quality-weightings’ to the benefits of health spending, to account for variation in the quality of health services utilised;
3. Document the changing context and processes for implementing UHC-reforms in Indonesia.

## Methodological approach

**Fig. 1:**
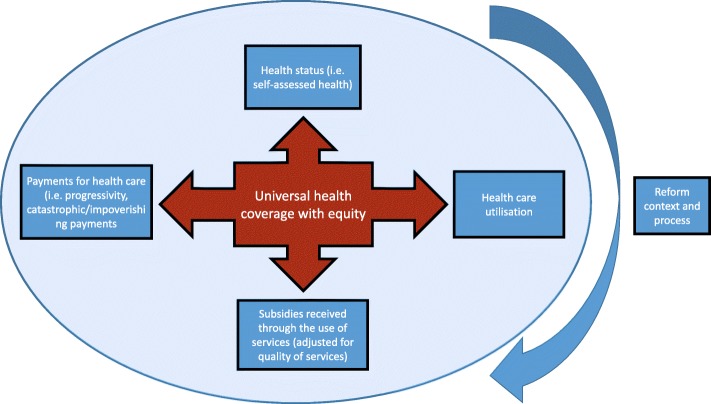
Methodological approach

Health equity research is typically concerned with four broad sets of outcomes: health care utilisation; subsidies received through the use of services; payments people make for health care (through for example, out-of-pocket payments, insurance premiums and direct and indirect taxes) [35, 36] and health status. In the case of health status, utilisation, and subsidies, the focus is on inequality, often defined as inequalities between the poor and the better-off [36]. In the case of health care payments, analysis tends to focus on progressivity (how much larger payments are as a share of income for the poor than for the better-off), the incidence of catastrophic payments (those that surpass a certain threshold), or the incidence of impoverishing payments (those that push a household over the poverty line). This methodological approach and associated outcomes to be measured in this study are summarised in Fig. 1.

The study will use a before and after design that employs both quantitative and qualitative methods. Outcomes will be evaluated at baseline (early 2018) and at end of target year of JKN full implementation (end 2019). The UHC reforms, consisting of multiple measures being progressed simultaneously over the next 2 years, will be evaluated as a ‘package’. While it will not be possible to draw conclusions concerning individual components, the study will disaggregate results by socioeconomic status, gender, levels of care and types of health care providers.

### Health care utilisation and distribution of health-care benefits (objective 1)

Benefit incidence analysis (BIA) measures the extent to which different groups benefit from public financing for health through their use of health services [37]. Operationalisation of the technique involves ranking the study population by a living standard measure, assessing the rate of utilisation of different health services, estimating the unit cost of each service, and multiplying the utilisation rates and unit costs to determine the amount of subsidy [38]. Direct payments by users are deducted before arriving at the final amount of government subsidy [38].

BIA requires data on health service utilisation, the cost of accessing health-care and socioeconomic status [15]. A cross-sectional household survey will be conducted at baseline and 18 months into implementation. Indonesia comprises approximately 17,000 islands divided into 34 provinces and 514 districts and municipalities [22]. The sampling for the ENHANCE household survey will be done in stages. First, a stratified sample of 10 provinces containing 74% of the population will be selected from 34 Indonesian provinces. Stratification of provinces will maximise representation of the population, capture the cultural and socioeconomic diversity, and be cost-effective to survey given the size and terrain of the country. At the next stage, two districts within each selected province will be purposively selected based on population density and fiscal capacity. From each district, two sub-districts and four villages (two villages per sub-district) will be chosen to ensure a mixed representation of rural and urban areas, and varying socio-economic status. Two enumeration areas (EAs) will then be selected from the villages (total of 80 EAs) using a nationally representative sample frame from the 2013 SUSENAS, a large-scale multi-purpose socioeconomic survey that covers a nationally representative sample typically composed of 200,000 Indonesian households [39]. Within each EA, field teams will randomly select 88 households based upon listings from the Central Bureau of Statistics to derive a final sample of 7040 households. In each selected household, one woman (the primary caregiver) or in her absence, the male head will be interviewed. The sample size will enable the determination of prevalence for characteristics with a 95% confidence interval and a precision of +/− 1%. Assuming that 12% of households [40] will exceed the threshold of 25% of total consumption expenditure on health (a commonly used indicator of payments for health that may have a catastrophic effect on household wellbeing [41]), we will be able to detect differences of 5% in characteristics between households that exceed the threshold and those that do not, with approximately 80% power and a type 1 error of 5.

Data will be collected electronically using laptops. An e-questionnaire will be designed using the NOVA Research Company’s Questionnaire Development System (QDS) 3.0 and administered with the computer-assisted personal interview (CAPI) program. The questionnaire will be piloted in selected EAs to test logistics and gather information to improve the quality and efficiency of the main survey. Field teams will be trained in e-data collection and administrative procedures including the content of the questionnaire, how to save completed interviews and how to transfer data to the Central Data Processing Centre for the study. National Health Accounts (NHA) will be used to estimate the unit cost of different health-care services, supplemented by Health Facility Costings [42]. NHA provide a detailed record of how Indonesia’s health resources are spent, on what services, and who pays for them. A critique of different national data sets for equity analysis in the health sector has been previously published [43].

The population will be ranked by the index and grouped into quintiles of equal size. Results will be presented in the form of bar charts indicating the relative share of total benefits received by socioeconomic quintiles. In addition, the distribution of benefits as depicted by the concentration curve (which plots the cumulative percentage of individuals ranked in ascending order of living standard against cumulative percentage of health-care utilisation or payment) will be compared against the 45° line of perfect equality [36, 38]. Dominance tests will be carried out to ascertain whether the differences are significant [36]. In addition to socioeconomic status, the distribution of health spending will also be explored by geographic location and by gender. The gender dimension of benefit from health spending is particularly important given the role of women as primary caregivers in times of illness or disability [44].

#### Socioeconomic status

The ENHANCE household survey will also collect information on household asset ownership to enable the construction of an asset index. This type of proxy measure of socio-economic status has been widely used by international development agencies such as the World Bank to assess and monitor health inequalities in LMICs [45]. The asset index will be constructed using principal component analysis [46] and based on a range of assets reflecting housing, utilities and livestock ownership.

### Distribution of the burden of paying for health-care (objective 1)

Financing incidence analysis (FIA), also known as progressivity analysis, will be used to assess how the burden of health financing is distributed in relation to household ability to pay (ATP) [47]. We will measure the progressivity of each individual source of financing and for the health financing system as a whole [47]. Financing sources are deemed progressive (regressive) if the rich contribute a relatively higher (lower) proportion of their income to health-care financing than the poor [48].

The 2012 National Socioeconomic Survey (SUSENAS) of Indonesia and the 2014 National Health Account (NHA) data will be used to estimate the baseline health-care financing mix and household contributions to health financing through direct and indirect taxation, out-of-pocket payments and payment of health insurance premiums. Evaluation in 2019 will use data from the 2016 NHA (available in early 2019) and the 2018SUSENAS. District Health Account Data (DHA), and other relevant cost data produced by BPS-Statistics will also be used for selected districts where appropriate. Tax thresholds and actual revenue generated through different forms of taxation will be obtained from the National Taxation Directorate and the Ministry of Finance and will in turn be triangulated with estimated tax revenue from the NHAs.

Progressivity of health care payments will be assessed by calculating the Kakwani Index [49], which is the difference between the concentration coefficient of health care payments and the Gini coefficient of household expenditure [47, 49]. The value of this index ranges from − 2 to 1 with a positive Kakwani index indicating that the health care financing system is progressive, or regressive if negative. A Kakwani index of zero indicates proportionality of health care payments [49]. The Kakwani Index will be calculated for each source of finance. The progressivity of the overall health financing system will be calculated by taking a weighted average of the Kakwani indices of the individual financing sources, where the weights are the shares of total revenues coming from each source.

#### Ability to pay

Adult equivalent consumption expenditure will be used as the measure of ability to pay. Consumption expenditure is generally considered a better measure of ability to pay than income in LMICs with a large informal sector, as consumption expenditure is smoothed over time and so better reflects long-term average well-being [50, 51]. For a detailed critique of different approaches to measuring ability to pay see O’Donnell et al. [36]. Household consumption expenditure will be translated into per adult equivalent household consumption, using the following formula:

AE = (A + αK)^*θ*^ Where A is the number of adults in the household, *θ* is the cost of children, K is the number of children and the degree of economies of scale [36, 51]. The values of *α* and *θ* were assumed to be 0.5 and 0.75, respectively [51, 52].

### Catastrophic and impoverishing health care payments (objective 1)

Out-of-pocket health expenditure exposes households to the risk of incurring large medical bills that can push households into financial catastrophe [53]. This is of major concern to countries such as Indonesia where more than 28 million people currently live below the poverty line and around 100 million remain vulnerable to falling into poverty, as their income hovers marginally above the national poverty line [54]. Measuring the catastrophic and impoverishing effects of out-of-pocket spending is therefore another important area of health equity research [36]. In line with other equity analyses [17, 53], households in this study will be considered to have incurred catastrophic health expenditure if the share of health expenditure in the household’s non-food expenditure is greater than a given threshold often around 25% [4] or within a range of 10 and 40% [54–56]. Indicators of catastrophic health expenditure will include catastrophic head count (share of households in the population whose health care costs expressed as a proportion of income exceed the threshold), catastrophic payment overshoot (average level by which payments, as a proportion of income, exceed the threshold) and the mean positive gap (payments in excess of the threshold average over all households) [36]. The data for this analysis will come from the 2013 SUSENAS Socioeconomic Survey conducted by the national Bureau of Statistics and the ENHANCE cross-sectional survey of Indonesian households (see section ii). Impoverishment will be assessed using both national and international poverty lines of US$1.90 and US$3.10 per day, respectively.

### Self-assessed health outcomes (objective 1)

While there is scepticism about the use of subjective health measures rather than more objective measures [57, 58], the former are much more readily available to researchers but more importantly, there exist robust findings of positive correlations between subjective assessments of health (SAH) and actual health and mortality [59, 60]. SAH has also been shown to be a good proxy for health service use in several countries [61]. The ENHANCE cross-sectional household survey (see section ii) will ask households to evaluate the general health condition of individual household members. A five-point scale with the following response options: ‘very good, good, fair, bad, and very bad’ will be piloted for use in this study [62]. SAH will be assessed at baseline in 2017 and 2 years into implementation in 2019. The measurement of SAH will be designed to enable comparison with existing measures used in other national health surveys in Indonesia such as the Basic Health Research Survey (Riskesdas). In addition to using SAH as one of the key outcome measures for this study, it will also be used in the BIA - whereby the distribution of benefits from using services will be compared with the distribution of the need for health care, using SAH as a proxy for need [38]. Several national surveys in LMICs include questions on SAH as proxies of health-care need [10].

#### Socio-economic status

As for the BIA, an asset index will be used to rank households according their socioeconomic status.

### Weighting the benefits of health spending to reflect quality of services (objective 2)

A recent systematic review of BIA studies in LMICs found that few studies account for variation in the quality of services received [10]. This is despite repeated calls for more precise measures of benefit/subsidy distribution that reflect the quality of services received [10, 37, 63].In this study benefits received by individuals will be weighted to reflect the quality of health services utilised, thereby providing a more precise measure of subsidy distribution. This is especially important in LMICs where it is recognised that the poor typically utilise lower quality health services compared to the rich [64]. The Institute of Medicine defines quality of care as the ‘degree to which health services for individuals and populations increase the likelihood of desired health outcomes and are consistent with current professional knowledge’ [65]. Measures of healthcare quality have been divided into 3 domains: structure or inputs to care, process or content of care, and outcomes of care [66]. According to Leslie and colleagues, each domain has its pros and cons: inputs are the necessary foundations for care but are not sufficient to describe its content or effects, process measures pertain directly to care delivery but are challenging to collect, and outcome measures assess the ultimate goal of the health system but reflect many factors beyond the health system itself [67]. Information on healthcare quality is sparse in LMICs and many analysts rely on standardised facility surveys that focus on inputs such as equipment, medicine supplies, and health workers [67–69]. A recent review of 8500 quality indicators used to assess performance-based financing programmes showed that over 90% measured structural aspects of quality [70]. For this study, data on the utilisation of different health facilities derived from our own cross-sectional household surveys (see objective 1a) will be linked to national health facility data on structural quality and staffing of public and private facilities. Two national surveys will be used: the PODES Infrastructure Census 2012 and the Health Facility Survey (Rifaskes) 2011. Scores for different structural quality domains will be derived from these national surveys and combined to develop a quality of care index from 0 to 1 for each facility.

### Understanding the context and process of implementing UHC-reforms in Indonesia (objective 3)

Document analysis and interviews will be used to understand the UHC policy adoption process. Specifically, we will develop a chronology of key events in the reform process and assess stakeholder support and political feasibility of the UHC-reforms [71, 72]. Key organisational and institutional documents from the Ministries of Health and Finance, local government planning and health offices, the private sector, the national social health insurance agency, and multilateral and bilateral agencies operating in Indonesia will be examined and interpreted in order to elicit meanings, gain understanding and develop empirical knowledge about the context within which UHC reforms have been pursued. In addition, in-depth interviews with approximately 15–20 key stakeholders will be conducted annually to understand the shifting power and positioning of different stakeholders around key elements of the UHC-reforms [72]. Stakeholders will be purposively sampled from Ministries of Health and Finance, health-care managers, professional associations, donors and private providers of health-care. Especially important will be the inclusion of members of the National Social Security Council (DJSN) which has legal authority to harmonise the JKN [73]. Interviewees will be chosen from two provinces facing distinctly different types of UHC implementation challenges including different levels of technical skills and management capabilities. These indicators will be obtained from the PODES Infrastructure Census 2012.

## Discussion

This study, evaluating pro-poor health care reforms in Indonesia, comes at an opportune time given the centrality of equity to the Sustainable Development Goals (SDGs). It will not only provide evidence on the equity-impact of Indonesia’s latest UHC-reforms but it will also help to advance metrics for UHC measurement. A variety of data sources (primary and secondary) are being pooled for this analysis. Drawing from a broader range of data will strengthen country estimates and better represent progress to UHC. Furthermore, this study will be one of the first to reflect the quality of services when calculating the distribution of public subsidies for health; an important methodological development in the field of health equity analysis. Taking account of the variation in the value of subsidies is especially important in countries such as Indonesia where around half of the population live in rural areas with limited access to skilled health workers and quality medicines. Moreover, like many other countries in the region Indonesia has a thriving private sector with two-thirds of health financing and more than half of all health services in private hands [74]. For the poor, this translates into high out-of-pocket payments that in turn limits access to health care and pushes many into poverty [1]. It may also place a disproportionate burden on them as they contribute a high proportion of their income towards health care financing compared to the rich. By taking a whole of system approach to the evaluation of UHC reforms, our financial and benefit incidence analyses will provide a comprehensive picture of the burden for paying for health services and the extent to which this ‘mixed’ public-private health system is meeting its equity goals. Also through our interviews with stakeholders we will gain insights into the political viability of the Indonesian UHC-reforms, an important but often neglected dimension of health system reform [72]. Apotential limitation of this study is that our cross-sectional household survey, designed to measure health care utilisation for the benefit incidence analysis, does not represent the entire population. We will empirically explore differences in health care utilisation between our sample and larger household surveys such as the Indonesian Demographic Health Survey (which collect less detailed utilisation data) to better understand the representativeness of our sample and generalisability of our findings. Finally, there is continued debate over the most useful and appropriate measures to assess the equity impact of UHC reforms. While this study measures a comprehensive suite of outcomes, such a detailed analysis will not be feasible, nor necessarily appropriate, for all health systems. We expect this study will help to prioritise outcome measures for assessing equity in health systems reform.

## Abbreviations

ATP: Ability to Pay
BIA: Benefit Incidence Analysis
BPJS: Badan Penyelenggara Jaminan Sosial (Social Insurance Administration Organization)
CAPI: Computer-Assisted Personal Interview
DHA: District Health Accounts
DJSN: Dewan Jaminan Sosial Nasional (National Social Security Council)
EA: Enumeration Area
FIA: Financing Incidence Analysis
GDP: Gross Domestic Product
GNI: Gross National Income
JKN: Jaminan Kesehatan Nasional (Indonesian national health insurance)
LMICs: Low and Middle Income Countries
MMR: Maternal Mortality Ratio
NHA: National Health Accounts
PODES: Potensi Desa (Infrastructure Supply Readiness Survey)
QDS: Questionnaire Development System
Rifaskes: Riset kesehatan dasar (Primary health care survey)
SDG: Sustainable Development Goals
SEG: Socio Economic Group
SHA: Self-Assessed Health
SUSENAS: Socioeconomic Survey
UHC: Universal Health Coverage
